# Supplementary material for: Structural basis of anti-SARS-CoV-2 activity of HCQ: specific binding to N protein to disrupt its interaction with nucleic acids and LLPS
Source: QRB Discov. 2021 Nov 29;2:e13. doi: 10.1017/qrd.2021.12 (PMC10392676; doi:10.1017/qrd.2021.12)
Supplement: Supplementary file 1 [file S2633289221000120sup001.pdf]

Supplementary Materials

Table S1
Figure S1-S7

Table S1. Residue-specific dissociation constants (Kd) of NTD and CTD residues.

NTD Residues	Kd (μM)	Error	CTD Residues	Kd (μM)	Error
**W52**	115.2	21.3	**281Q**	42.9	10.1
**L56**	127.1	24.9	**282T**	41.4	5.4
**H59**	125.8	30.2	**325T**	78.9	22.6
**S79**	86.1	21.4	**329T**	75.5	16.8
**G147**	164.4	40.2	**330W**	67.9	15.1
**I157**	85.8	15.5	**336A**	35.1	20.9
**V158**	136.1	20.3	**337I**	58.0	12.3
**L159**	117.1	28.2			
**T166**	45.6	15.6			
**A173**	133.3	21.9			
**E174**	97.3	19.1			
**Average**	**112.1**	**32.2**	**Average**	**57.1**	**17.6**

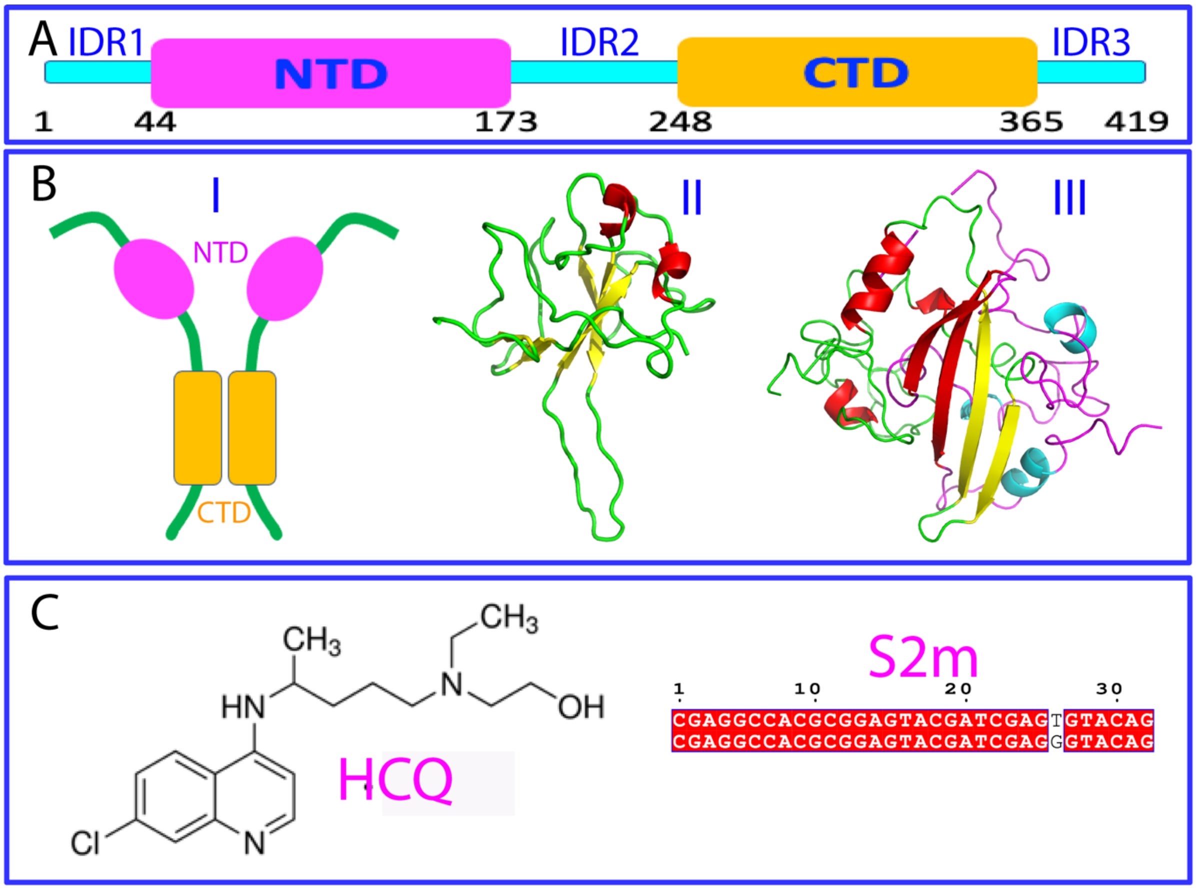

Figure S1. Structures of SARS-CoV-2 N protein, HCQ and S2m.

(A) Domain organization of SARS-CoV-2 N protein. (B) Schematic representation of the dimeric N protein (I), three-dimensional structures of NTD (II) and dimeric CTD (III). (C) Chemical structures of hydroxychloroquine (HCQ) and sequences of 32-mer ssDNA S2m of SARS-Cov-1 (upper) and SARS-CoV-2 (lower).

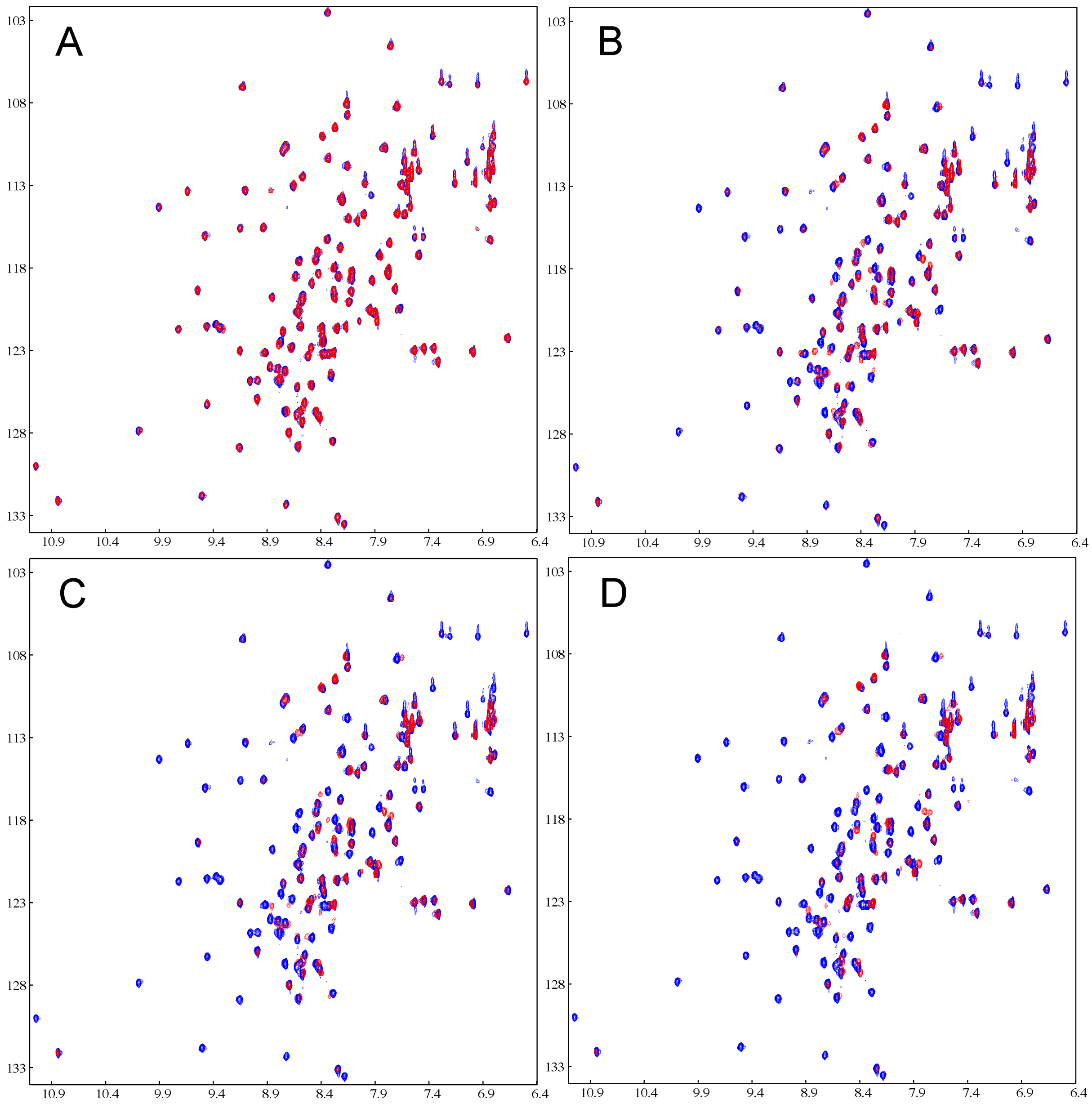

Figure S2. S2m binds NTD of SARS-CoV-2 N protein.
Superimposition of HSQC spectra of NTD at 100 μM in the free state (blue) and in the presence of S2m (red) at 1:0.1 (A), 1:0.5 (B), 1:1.0 (C) and 1:2.5 (D) (NTD:S2m).

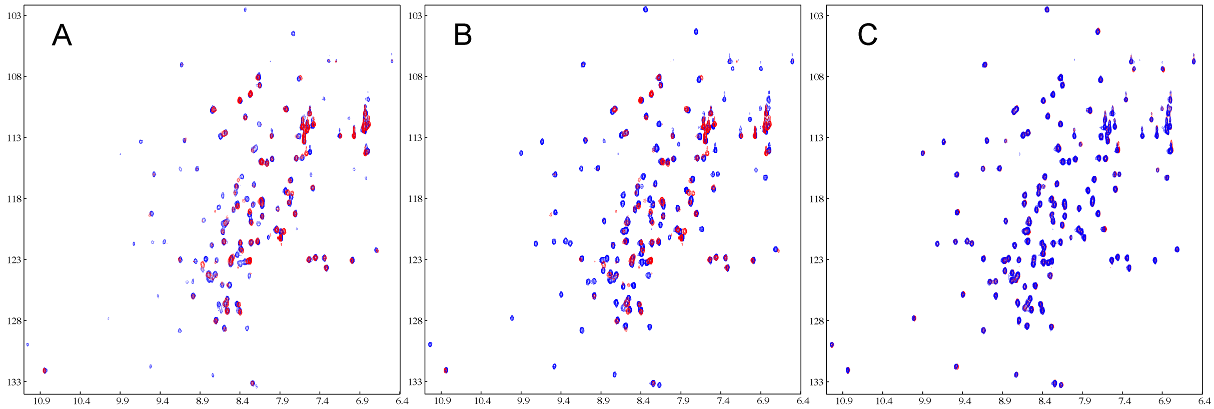

Figure S3. HCQ displaces S2m from binding with NTD of SARS-CoV-2 N protein. Superimposition of HSQC spectra of NTD at 100 μM in the presence of S2m at 1:2.5 (NTD:S2m) (red) and with additional addition of HCQ (blue) at 3.75 (A) and 1:15 (B). (C) Superimposition of HSQC spectra of NTD at 100 μM in the presence of only HCQ at 1:15 (blue) and in the presence of both S2m at 1:2.5 and HCQ at 1:15 (red).

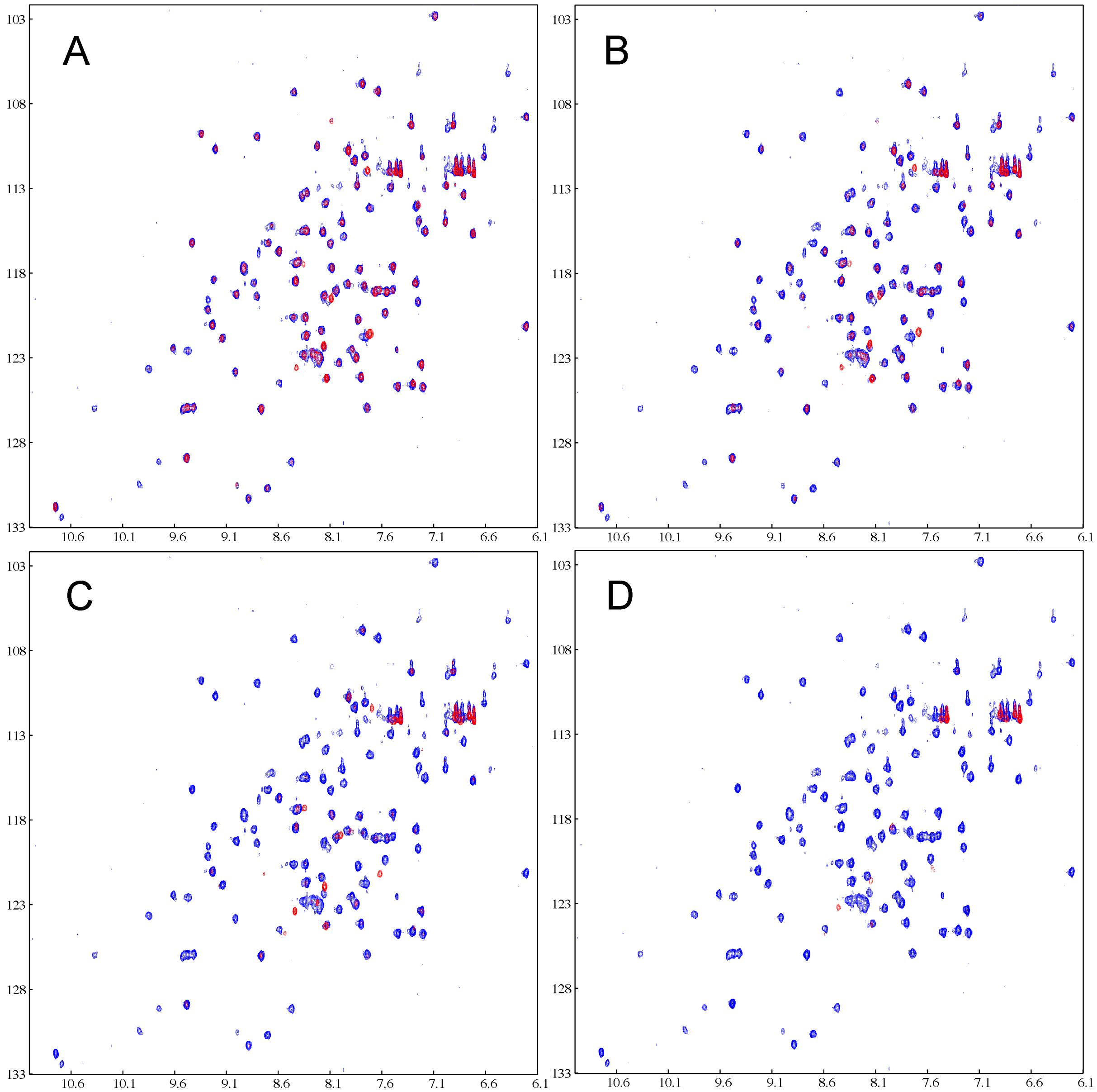

Figure S4. S2m binds CTD of SARS-CoV-2 N protein.
Superimposition of HSQC spectra of CTD at 200 μM in the free state (blue) and in the presence of S2m (red) at 0.05 (A), 0.1 (B), 0.5 (C) and 1.0 (D).

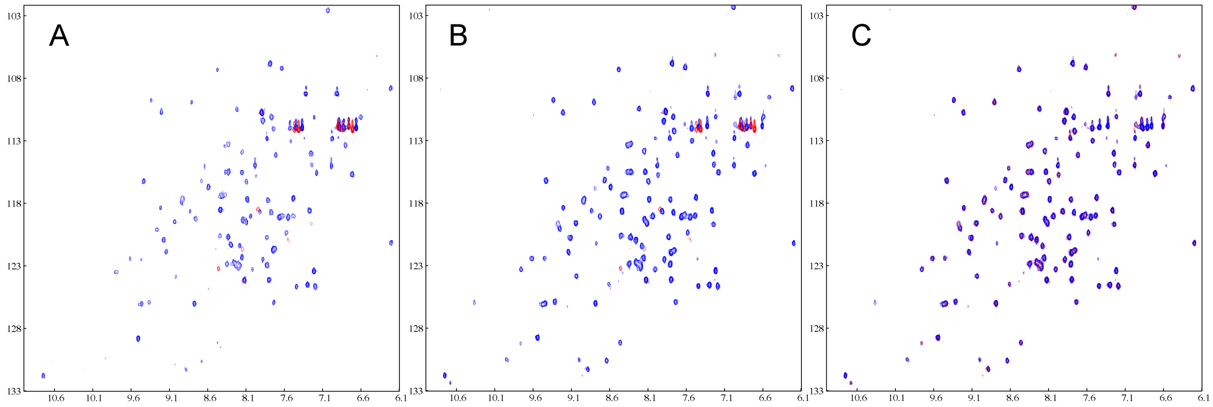

Figure S5. HCQ displaces S2m from binding with CTD of SARS-CoV-2 N protein. Superimposition of HSQC spectra of CTD at 200 μM in the presence of S2m at 1:1 (CTD:S2m) (red) and with additional addition of HCQ (blue) at 1:88 (A) and 1:7.5 (B). (C) Superimposition of HSQC spectra of CTD at 200 μM in the presence of only HCQ at 1:7.5 (blue) and in the presence of both S2m at 1:1 and HCQ at 1:7.5 (red).

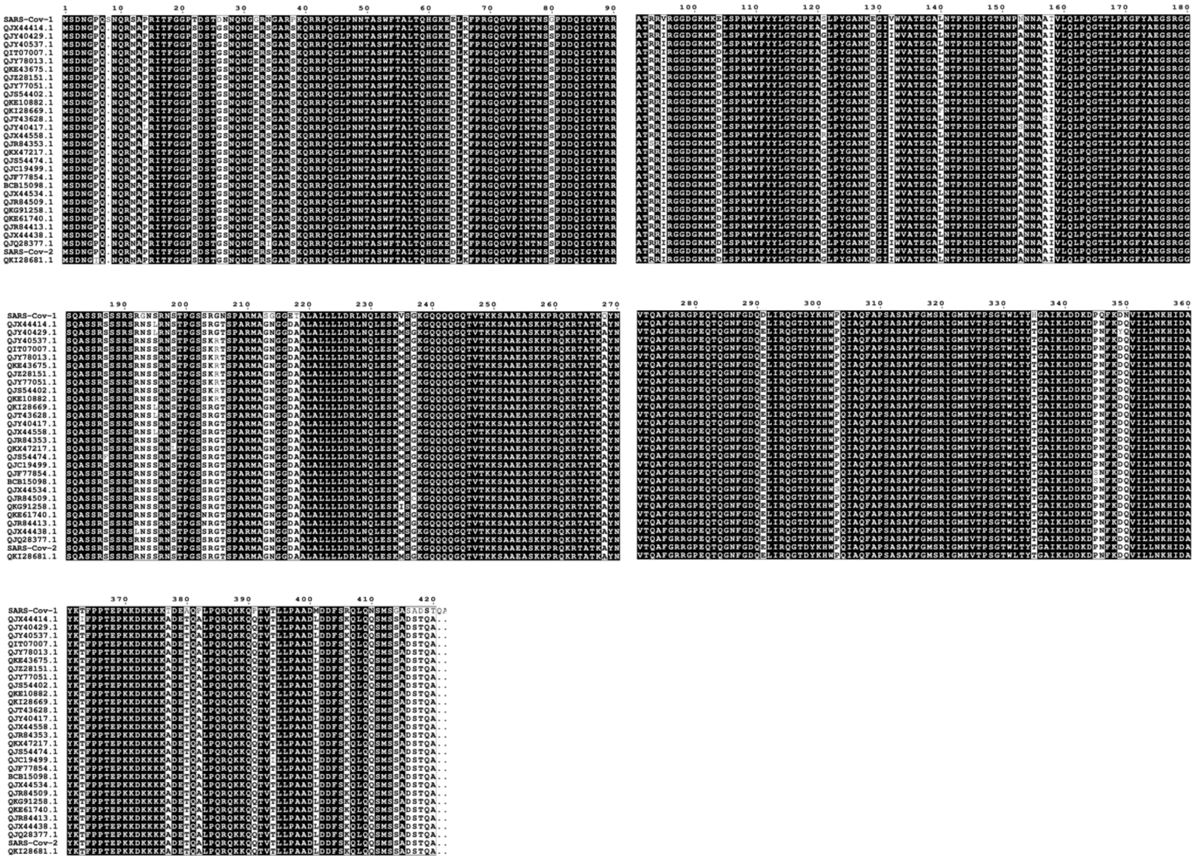

Figure S6. Sequence alignment of nucleocapsid (N) proteins of SARS-CoV-1 as well as SARS-CoV-2 and its major variants.

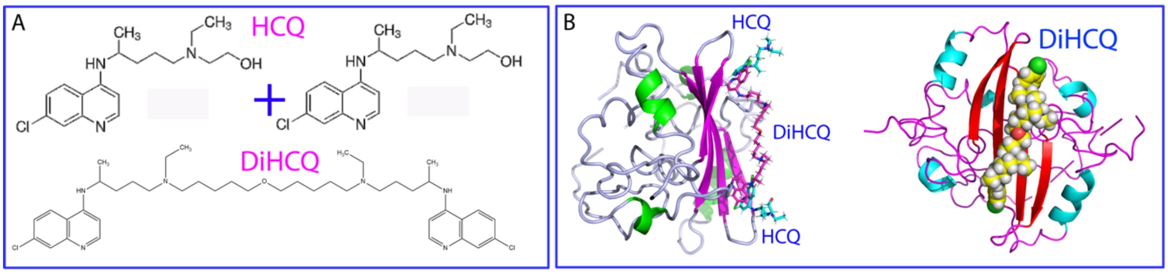

Figure S7. A proposed strategy to design better anti-SARS-CoV-2 molecule from HCQ with the higher affinity and specificity.
(A) Schematic representation of linking two HCQ molecules to form DiHCQ. (B) Docking structures of the dimeric CTD in complex with HCQ or with DiHCQ with HCQ/DiHCQ displayed in stick or sphere respectively.
